# Left Ventricular End Diastolic Volume and Ejection Fraction Calculation: Correlation between Three Echocardiographic Methods

**DOI:** 10.1155/2020/8076582

**Published:** 2020-02-28

**Authors:** Nadia Benyounes, Clélie Van Der Vynckt, Thierry Tibi, Alexandra Iglesias, Olivier Gout, Sylvie Lang, Laurence Salomon

**Affiliations:** ^1^Cardiology Unit, A. de Rothschild Foundation, Paris, France; ^2^Alsacienne School, Paris, France; ^3^Department of Neurology, A. de Rothschild Foundation, Paris, France; ^4^Department of Cardiology, Saint-Antoine Hospital, Paris, France; ^5^Clinical Research Department, A. de Rothschild Foundation, Paris, France

## Abstract

**Background:**

Left ventricular ejection fraction (LVEF) and end diastolic volume (EDV) are measured using Simpson's biplane (SB), 3-dimensional method (3DE), and speckle tracking (STE). Comparisons between methods in routine practice are limited. Our purpose was to compare and to determine the correlations between these three methods in clinical setting.

**Methods:**

LVEF and EDV were measured by three methods in 474 consecutive patients and compared using multiple Bland–Altman (BA) plots. The correlations (R) between methods were calculated.

**Results:**

Median (IQR) LVEF_SB, LVEF_STE, and LVEF_3DE were 63.0% (60–69)%, 61% (57–65)%, and 62% (57–68)%. Median (IQR) EDV_SB, EDV_STE, and EDV_3DE were 85 ml (71–106) ml, 82 ml (69–100) ml, and 73 ml (59–89) ml. R between LVEF_SB and LVEF_3DE was 0.65 when echogenicity was good and 0.43 when poor. R for EDV_SB and EDV_3DE was 0.75 when echogenicity was good and 0.45 when poor. On BA analysis, biases were acceptable (<3.5% for LVEF) but limits of agreement (LOA) were large: 95% of the differences were between −15.4% and +18.8% for LVEF as evaluated by SB in comparison with 3DE, with a bias of 1.7%. In the comparison EDV_SB and EDV_3DE, the bias was 14 ml and the LOA were between −24 ml and +53 ml. On linear regressions, LVEF_3DE = 17.92 + 0.69 LVEF_SB and EDV_3DE = 18.94 + 0.63 EDV_SB.

**Conclusions:**

The three methods were feasible and led to acceptable bias but large LOA. Although these methods are not interchangeable, our results allow 3DE value prediction from SB, the most commonly used method.

## 1. Introduction

Transthoracic echocardiography (TTE) is the most commonly used diagnostic tool for left ventricular (LV) systolic dysfunction. The new guidelines state that LV systolic function should be routinely assessed using two-dimensional echocardiography (2DE) or three-dimensional echocardiography (3DE) [1]. In laboratories with experience in 3DE, 3DE measurement and reporting of LV volumes are recommended. During, and after cancer therapy, it is recommended to calculate LVEF with the best available method in the laboratory and ideally 3DE [2]. The SB method is time-consuming and prone to intraobserver and interobserver variability [3]. New techniques like speckle tracking echocardiography (STE) are available. STE enables LVEF and EDV measurement. Tracking-based EF assessment has been proved to be fast and feasible, with lower interobserver and intraobserver variability [4]. LVEF and volumes depend on the imaging modality that is used [5].

Few data exist regarding the agreement between LVEF and EDV as determined by these different methods, in clinical practice.

We aimed to assess the impact of the method used on LVEF and LVED values as part of a routine practice and to establish correlations between these three methods.

## 2. Materials and Methods

The study population comprised consecutive patients undergoing TTE at the A. de Rothschild Foundation Hospital, Paris, France, between March 2015 and August 2016. TTEs were performed by an expert cardiologist. Hospitalized patients comprised approximately 90% of our population.

### 2.1. Transthoracic Echocardiographies

TTEs were performed using a commercially available ultrasound system (EPIQ7, version 1.4.1, Philips Ultrasound) with a 1.3–4.2 MHz phased array transducer.

LVEF and EDV were calculated by the three methods almost simultaneously, in a predefined sequence, in all patients: Simpson's biplane method, 3DE, and STE, were performed according to current guidelines [1].

Simpson's biplane method: LVEF was calculated using the manual tracing on apical four and apical two-chamber views.

Speckle tracking method: the three apical views (four-, two-, and three-chamber) were recorded with a frame rate between 70 Hz and 80 Hz. Careful manual tracking of the endocardial contour was performed. Myocardial deformation and LVEF were assessed in a semiautomatic manner, based on greyscale images.

Three-dimensional echocardiography: LVEF and EDV measurements were performed after a 3D image acquisition including the entire LV within the pyramidal data set (4 beats volume acquisition). The 3D-guided biplane analysis was the method used. The different steps were described elsewhere [6].

Patient's echogenicity was reported as good, moderate, or poor, to allow further analysis by “acoustic window” subgroups. The quality of the acoustic window had to be sufficient to allow the calculation of LVEF and EDV by at least two of the three methods. Otherwise, the TTE was excluded.

As the aim of this study was to compare three echocardiographic methods for LVEF and LVED measurement as part of an everyday practice, no rereading by a second observer was done.

### 2.2. Statistical Analysis

Pearson's correlation coefficient was used to analyse correlations between LVEFs and EDVs when calculated by two different methods.

As SB is the most available method, simple linear regression was used to assess the relationship between LVEF_SB and LVEF_3DE or LVEF_STE, and between EDV_SB and EDV_3DE or EDV_STE.

Analysis of variance was used to compare the three LVEF means (mean LVEF_SB, mean LVEF_3DE, and mean LVEF_STE) and the three EDV means.

Bland–Altman analysis, in which the mean of two measurements was plotted against the difference, was used to measure the variability between two techniques. Differences between methods are in absolute units.

## 3. Results and Discussion

### 3.1. Results

#### 3.1.1. Population and Echocardiographic Characteristics

Among the consecutive TTEs performed during the study period, 39 were excluded because of atrial fibrillation and an additional 65 because more than one LVEF and/or EDV methods of measurement were not performed.

Consequently, the study involved 474 TTEs. The main patients' clinical and echocardiographic characteristics are shown in Table 1.

The indications for TTE were as follows: ischaemic stroke or transient ischaemic attack and haemorrhagic stroke (*n* = 275), occlusion of the central retinal artery (*n* = 12), chest pain (*n* = 12), cancer therapy monitoring (*n* = 12), dyspnoea (*n* = 11), hypertension (*n* = 19), diabetes mellitus (*n* = 11), heart murmur exploration and control of valvular disease, endocarditis and suspicion of endocarditis (*n* = 28), preoperative assessment (*n* = 10), LVEF evaluation and/or heart failure (*n* = 5), pulmonary embolism (*n* = 3), subarachnoid haemorrhage (*n* = 3), ischaemic heart disease (*n* = 5), history of AF (*n* = 4), syncope (*n* = 5), and other indications (*n* = 59).

#### 3.1.2. Echocardiographic Findings: LVEF and EDV

LVEF_SB, LVEF_STE, and LVEF_3DE measurements are displayed in Table 2.

Analysis of variance (ANOVA) revealed significant differences between the average LVEF_SB and LVEF_3DE and between the average LVEF_SB and LVEF_STE, with *p* values of, respectively, 0.005 and <0.0001.

**Figure 1:**
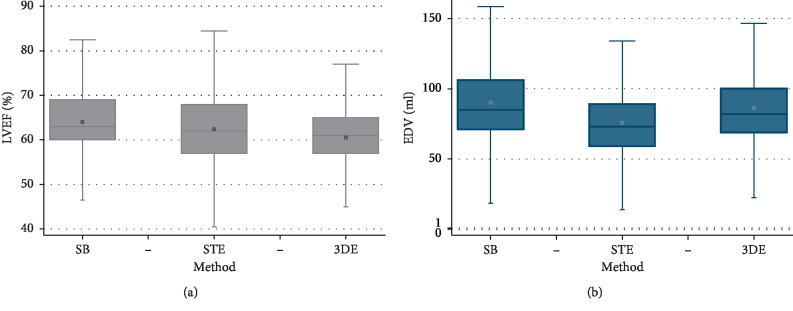
LVEF (a) and EDV (b) values according to the method used. Box-plot representation showing the distribution of LVEF values according to the method which was used. The inbox line represents the mid value. The inbox circle represents the mean value. The edges of the box represent the 25th and 75th percentiles (*Q*1 and *Q*3), and the ends of the whiskers represent the upper and lower adjacent values, which are the most extreme values within *Q*3 + 1.5 *∗* (*Q*3 − *Q*1) and *Q*1 − 1.5 *∗* (*Q*3 − *Q*1), respectively. At first sight, 3DE values seem to be the lowest. Box-plot representation showing the distribution of EDV values according to the method which was used. At first sight, STE values seem to be the lowest.

The modified Simpson's rule produced higher LVEFs than STE and 3DE, as shown in Figure 1(a). Three-dimensional echocardiography provided the lowest LVEF.

Median EDV_SB, EDV_STE, and EDV_3DE are shown in Table 2.

ANOVA revealed significant differences between the average EDV_SB and EDV_3DE and between the average EDV_SB and EDV_STE, with *p* values of, respectively, <0.0001 and *p*=0.04.

The modified Simpson's rule produced higher EDVs than STE and 3DE, as shown in Figure 1(b). Speckle tracking echocardiography provided the lowest EDV.

The highest average EDV was provided by the modified Simpson's rule and the lowest by three-dimensional echocardiography (Figure 1(b)).

#### 3.1.3. Echocardiographic Findings: Correlations between Methods

Among the three echocardiographic LVEF estimates, SB and 3DE assessments had the best correlation (*r* = 0.62, *n* = 450, *p* < 0.0001). Pearson's coefficients for the correlations LVEF_SB vs LVEF_STE and LVEF_3DE vs LVEF_STE were, respectively (*r* = 0.56, *n* = 469, *p* < 0.0001 and *r* = 0.45, *n* = 445, *p* < 0.0001).  Table 3 shows Pearson's coefficients for the correlations between the three echocardiographic LVEF estimates.

Patient's echogenicity influenced these correlations. When echogenicity was good, Pearson's coefficients for the correlations LVEF_SB vs LVEF_3DE, LVEF_SB vs LVEF_STE, and LVEF_3DE vs LVEF_STE improved. They reached, respectively, *r* = 0.65, *n* = 264, *p* < 0.0001; *r* = 0.60, *n* = 269, *p* < 0.0001; and *r* = 0.49, *n* = 261, *p* < 0.0001.

When echogenicity was moderate, Pearson's coefficients for the correlations LVEF_SB vs LVEF_3DE, LVEF_SB vs LVEF_STE, and LVEF_3DE vs LVEF_STE were, respectively, *r* = 0.58, *n* = 169, *p* < 0.0001; *r* = 0.53, *n* = 177, *p* < 0.0001; and *r* = 0.44, *n* = 167, *p* < 0.0001.

In patients with poor echogenicity, correlation coefficients were not significant and decreased to, respectively, *r* = 0.43, *n* = 17, *p*=0.0825; *r* = 0.35, *n* = 23, *p*=0.0969; and *r* = 0.18, *n* = 17, *p*=0.4835.

Correlations between the three echocardiographic EDV estimates were measured and appeared to be better than those of LVEF comparisons. For EDV_SB vs EDV_3DE, EDV_SB vs EDV_STE, and EDV_3D vs EDV_STE, Pearson's coefficients were, respectively, *r* = 0.76, *n* = 452, *p* < 0.0001; *r* = 0.88, *n* = 468, *p* < 0.0001; and *r* = 0.72, *n* = 448, *p* < 0.0001.

The correlations were quite similar when echogenicity was good or moderate and altered when echogenicity was poor. Hence, Pearson's correlation coefficients for EDV_SB vs EDV_3DE, EDV_SB vs EDV_STE, and EDV_3D vs EDV_STE were, respectively, *r* = 0.75 (*p* < 0.00001), *r* = 0.88 (*p* < 0.00001), and *r* = 0.71 (*p* < 0.00001) when echogenicity was good, and, respectively, *r* = 0.80 (*p* < 0.00001), *r* = 0.90 (*p* < 0.00001), and *r* = 0.76 (*p* < 0.00001) when echogenicity was moderate.

When echogenicity was poor, correlation coefficients decreased and reached, respectively, *r* = 0.45 (*p*=0.0674), *r* = 0.65 (*p*=0.0009), and *r* = 0.35 (*p*=0.1736).

**Figure 2:**
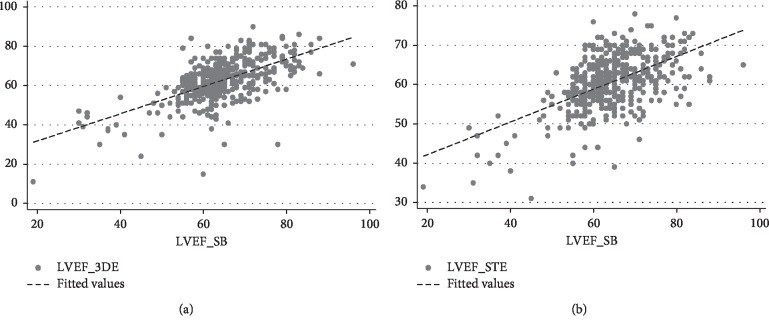
Simple linear regression analysis between LVEF_3DE and LVEF_SB (a) and LVEF_STE and LVEF_SB (b). LVEF_3DE = 17.92 + 0.69 LVEF_SB. LVEF_STE = 33.84 + 0.42 LVEF_SB.

Linear regressions were used to describe the relationships between the LVEF and EDV estimates from the different methods.  Figure 2 depicts the simple linear regression analysis between LVEF_3DE and LVEF_SB (Figure 2(a)) and between LVEF_STE and LVEF_SB (Figure 2(b)) in the entire series.

For one unit increase in LVEF_SB, a 0.69 unit increase in LVEF_3DE and a 0.42 increase in LVEF_STE are expected (Figure 2).

**Figure 3:**
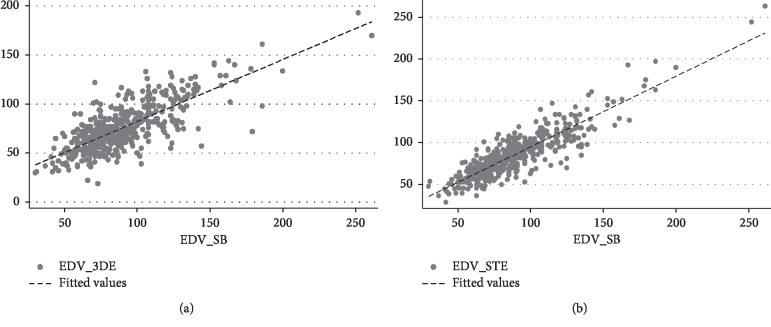
Simple linear regression analysis between EDV_3DE and EDV_SB (a) and EDV_STE and EDV_SB (b). EDV_3DE = 18.94 + 0.63 EDV_SB. EDV_STE = 10.51 + 0.84 EDV_SB.

Figure 3 depicts the simple linear regression analysis between EDV_3DE and EDV_SB (Figure 3(a)) and between EDV_STE and EDV_SB (Figure 3(b)) in the entire series.

For one unit increase in EDV_SB, a 0.63 unit increase in EDV_3DE and a 0.84 increase in EDV_STE are expected (Figure 3).

After excluding the 23 TTEs with poor echogenicity, the regression parameters were quite similar in the two cases.

#### 3.1.4. Echocardiographic Findings: The Variability between Methods

**Figure 4:**
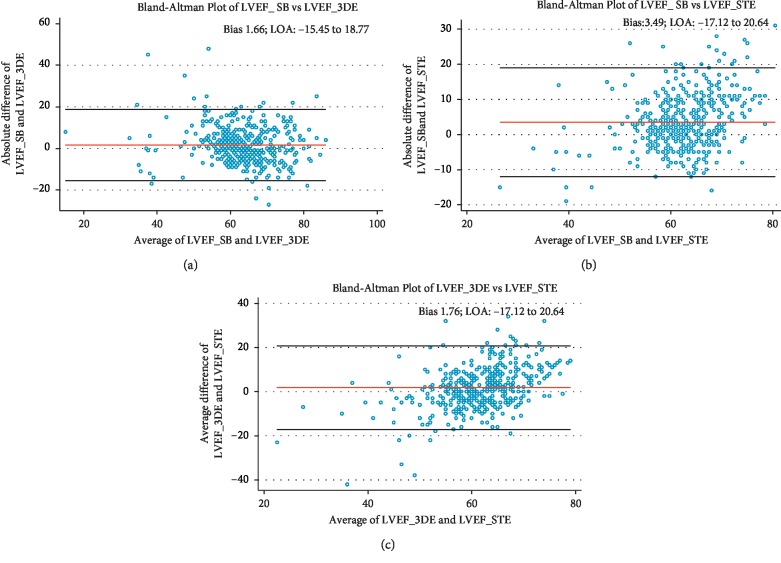
Bland–Altman plots for the comparisons between (a) LVEF_SB and LVEF_3DE, (b) LVEF_SB and LVEF_STE, and (c) LVEF_3DE and LVEF_STE. Bias: 1.66% (95% CI 0.86–2.47); bias: 3.49% (95% CI 2.77–4.20); and bias: 1.76% (95% CI 0.86–2.65), respectively.

**Figure 5:**
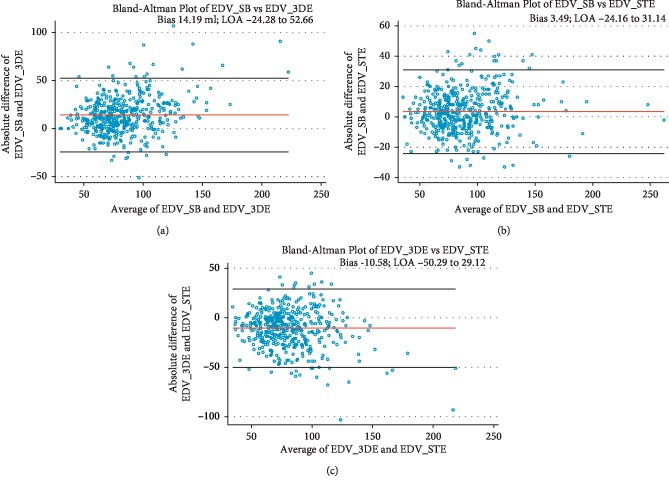
Bland–Altman plots for the comparisons between (a) EDV_SB and EDV_3DE, (b) EDV_SB and EDV_STE, and (c) EDV_3DE and EDV_STE. Bias: 14.19 ml (95% CI 12.38–16.00); bias: 3.49 ml (95% CI 2.23–4.75); and Bias: 10.58 ml (95% CI -12.46 to -8.71), respectively.

Bland–Altman analysis was used to evaluate the variability between LVEF_3DE and LVEF_SB, LVEF_STE and LVEF_SB (Figure 4), EDV_3DE and EDV_SB, and EDV_STE and EDV_SB (Figure 5). Figures 4 and 5 show the values of the mean differences (bias) and the limits of agreement (LOA).

Patients with normal LVEF using SB (≥50%) and misclassified by one of the two other methods were rare. Only 26 of these patients (5.8%) had LVEF <50% using 3DE or STE. Twenty patients were misclassified by 3DE (4.5%), 8 patients by STE (1.8%), and 2 patients by the two methods (0.5%).

When the limit was set at 45% using SB, 16 patients (3.6%) were misclassified by one of the two other methods.

### 3.2. Discussion

This is a prospective study to compare three approaches of LVEF and EDV estimation as part of a routine echocardiographic practice. Well-designed trials are indeed essential. Although real life practice may sometimes be different, it also deserves to be explored [7, 8].

Today, the modified Simpson's biplane rule is the most commonly used echocardiographic technique for LVEF assessment. However, recent evolutions in echocardiography have led to new techniques, which include STE and 3DE. STE-based LVEF has been shown to be feasible, accurate, and reproducible, with lower interobserver and intraobserver variability than other echocardiographic and magnetic resonance imaging methods [4].

Three-dimensional echocardiography has become the recommended technique for repeated LVEF measurements during, and after cancer therapy, when available [9].

However, for now, only top of the line commercial ultrasound systems are equipped with 3DE and STE software. Hence, this study which looks at an intraindividual comparison of these three echocardiographic methods provides useful data.

Our results support the feasibility of these three methods during routine practice, since only 12% of the patients in sinus rhythm explored during TTE did not have at least two of the three methods done.

In this study, the mean LVEFs and EDVs were significantly different. These results could be challenged, since at least one study shows no differences between the means (ANOVA) of LVEFs as measured by four different techniques (tracking-based LVEF, visual interpretation of all the three apical views, biplane EF using the modified Simpson's rule, and cardiac MRI) [4]. This study involved 75 patients who had both echocardiography and MRI. In fact, this is thoroughly discussed in the article; the study's setting was very far from real-life conditions, with, as stated, “rather strict conditions.”

In our study, Simpson's biplane method provided the highest mean LVEF and the highest mean EDV, while 3DE provided the lowest mean LVEF. Three-dimensional echocardiography significantly underestimated EDV, which is in line with previous studies on patients with rather preserved LVEF [10], on patients with left ventricular aneurysm [11], and on patients with ischaemic and dilated cardiomyopathy [12]. In the latest, it was hypothesized that this underestimation could be due to difficulties in imaging the entire left ventricle in the 3D pyramidal volume.

In our study, Bland–Altman analysis provided low biases between methods for LVEF measurements, all being less than 5% in absolute units: 1.7% between SB and 3DE, 1.8% between 3DE and STE, and 3.5% between SB and STE. This might be considered clinically acceptable.

A recent study in patients with LV dysfunction reported such low variability in biases between echocardiographic modalities for LVEF measurement. The mean absolute differences between LVEF as determined by quantitative vs visual echocardiographic methods were all less than 5% [9].

The biases were also low for EDV measurements in our study: 14 ml between SB and 3DE, 3.5 ml between EDV_SB and EDV_STE, and −11 ml between EDV_3DE and EDV_STE.

Despite the documented acceptable biases, the huge LOA precludes routine interchangeability between methods in such clinical setting, unless adjustments are made.

This systematic intermodality variability for LVEF and EDV measurements in our study is concordant with previous reports [5].

The correlations between methods were moderate (see Table 3), even depending on echogenicity. Pearson's coefficients for the correlations LVEF_SB vs LVEF_3D, LVEF_SB vs LVEF_STE, and LVEF_3DE vs LVEF_STE were, respectively, *r* = 0.62, *p* < 0.00001; *r* = 0.56, *p* < 0.00001; and *r* = 0.45, *p* < 0.00001. A recent study reported similar moderate correlations between different methods for LVEF measurements [9]. The correlations, in this study and others [9], depend on echogenicity. In ours, Pearson's coefficients for correlations were not significant when echogenicity was poor. This is a major issue in echocardiography, to avoid measurements that could be false, due to insufficient echogenicity. The operators performed the echocardiographic methods in only 23 patients with poor echogenicity. This once again reflects real-life conditions.

Of note, echogenicity is often worse in hospitalized patients than in outpatients. Indeed, only 57% of the scans in our study had good echogenicity.

This seems usual in such cohorts. In a previous echocardiographic study, good echogenicity was reported in only 48.1% of scans [8], and in a more recent one, excellent echogenicity was reported in 3.6% of scans and good echogenicity in 40.6% [9].

Finally, we could obtain, from 474 scans, interesting linear regression analyses, allowing the prediction of LVEF_3D and EDV_3D, from LVEF_SB and EDV_SB: LVEF_3DE = 17.93 + 0.69 LVEF_SB and EDV_3DE = 18.05 + 0.64 EDV_SB.

It is of major importance to emphasize that we do absolutely not suggest the replacement of 3DE by 2DE measurements. We only provide a tool to approach 3D LVEF and EDV, when 3D software is not available on the echo machine.

## 4. Study Limitations

All the measurements were performed online, without rereading by a second observer. However, this was part of the underlying philosophy of this study, which aimed to assess, in consecutive patients in our echo lab real-life practice, the feasibility of the three echocardiographic methods for LVEF and EDV assessment and the correlations between methods.

Our results might only be true with the equipment used in this study (EPIQ7, version 1.4.1, Philips Ultrasound) and might be slightly different on more recent machines.

## 5. Conclusions

This study was exclusively driven by a practical goal of improving the assessment of LVEF and EDV in routine.

The use of three echocardiographic methods (SB, 3DE, and STE) is confirmed to be possible in everyday practice.

The variability in LVEF measurements did not exceed 5% and was hence clinically acceptable.

When close echocardiographic monitoring is needed, longitudinal assessments of a given patient should ideally be accomplished using a single echocardiographic modality, on a single machine. This is actually very difficult and sometimes impossible in real life. Hence, there may be some room for the kind of formulas we report. The prediction of 3-dimensional LVEF and EDV from biplane ones is possible, even if in no way one modality can replace another.

This may be valuable when 3DE machines are not available.

## Figures and Tables

**Table 1:** Main clinical and echocardiographic characteristics of the patients in this study.

Characteristics	(*n* = 474)
Age (years)	63 (50–75)
Men	256 (54.0%)
LVM indexed to body surface (g/m^2^)	93 (77–111)
LVEF (%)	
Median, SB	63 (60–69)
Median, 3D	62 (57–68)
Median, STE	61 (57–65)
EDV (ml)	
Median, SB	85 (71–106)
Median, 3D	73 (59–89)
Median, STE	82 (69–100)
GLS (%)	-19 (-21–18)
Echogenicity	
Good	272 (57.4%)
Moderate	179 (37.8%)
Poor	23 (4.9%)

Data are median and interquartile range or number (%). GLS: global longitudinal strain; LVEF: left ventricular ejection fraction; EDV: end diastolic volume; SB: Simpson's biplane method; 3D: three-dimensional echocardiography; STE: speckle tracking echocardiography; LVM: left ventricular mass.

**Table 2:** Mean LVEFs and EDVs obtained by the different methods.

LVEF (%)	*n*	Mean ± SD (%)	(95% CI)	*p* ^*∗*^

SB	474	64 ± 9	(63–65)	
3DE	450	62 ± 10	(61–63)	≤0.005
STE	469	61 ± 7	(60–61)	

EDV (ml)	*n*	Mean ± SD (%)	(95% CI)	*p* ^*∗*^

SB	473	90 ± 29	(87–93)	
3DE	450	76 ± 25	(74–78)	≤0.04
STE	469	86 ± 28	(84–89)	

^*∗*^ANOVA.

**Table 3:** Pearson's coefficients for the correlations between the three echocardiographic LVEF estimates.

LVEF (%)	SB	3DE	STE
SB	1.0000		
3DE	0.6153^*∗*^ (*n* = 450)	1.0000	
STE	0.5550^*∗*^ (*n* = 469)	0.4503 ^*∗*^ (*n* = 445)	1.0000

^*∗*^ *p* < 0.0001.

## Data Availability

The data used to support the findings of this study can be obtained from the corresponding author.
